# Resistance to Dopamine Agonists in Pituitary Tumors: Molecular Mechanisms

**DOI:** 10.3389/fendo.2021.791633

**Published:** 2022-01-12

**Authors:** Claudia Pivonello, Roberta Patalano, Mariarosaria Negri, Rosa Pirchio, Annamaria Colao, Rosario Pivonello, Renata Simona Auriemma

**Affiliations:** ^1^ Dipartimento di Medicina Clinica e Chirurgia, Sezione di Endocrinologia, Università di Napoli (Federico II), Naples, Italy; ^2^ Dipartimento di Sanità Pubblica, Università di Napoli (Federico II), Naples, Italy; ^3^ United Nations Educational, Scientific and Cultural Organization (UNESCO) Chair for Health Education and Sustainable Development, Federico II University, Naples, Italy

**Keywords:** dopamine, dopamine agonist, pituitary tumors, cabergoline, prolactinomas

## Abstract

Pituitary neuroendocrine tumors (PitNET) are commonly benign tumors accounting for 10-25% of intracranial tumors. Prolactin-secreting adenomas represent the most predominant type of all PitNET and for this subtype of tumors, the medical therapy relies on the use of dopamine agonists (DAs). DAs yield an excellent therapeutic response in reducing tumor size and hormonal secretion targeting the dopamine receptor type 2 (D2DR) whose higher expression in prolactin-secreting adenomas compared to other PitNET is now well established. Moreover, although DAs therapy does not represent the first-line therapy for other PitNET, off-label use of DAs is considered in PitNET expressing D2DR. Nevertheless, DAs primary or secondary resistance, occurring in a subset of patients, may involve several molecular mechanisms, presently not fully elucidated. Dopamine receptors (DRs) expression is a prerequisite for a proper DA function in PitNET and several molecular events may negatively modify DR membrane expression, through the DRs down-regulation and intracellular trafficking, and DR signal transduction pathway. The current mini-review will summarise the presently known molecular events that underpin the unsuccessful therapy with DAs.

## Introduction

Dopamine agonists (DAs) are chemical compounds that, by directly binding to the dopamine receptors (DRs), induce biological actions resembling that of endogenous dopamine (1). In the context of pituitary neuroendocrine tumors (PitNET), DAs lead to the inhibition of hormonal secretion and tumor shrinkage in different pituitary tumor histotypes by mainly binding to the dopamine receptor type 2 (D2DR), largely expressed in lactotroph pituitary cells but also localized in other pituitary cell sub-populations including somatotroph and corticotroph cells (2).

DAs can be classified in ergot derivatives, including bromocriptine (BRC), cabergoline (CAB), pergolide and lisuride, and in non-ergot derivatives such as quinagolide. The most commonly used DAs as a medical treatment for PitNET are currently BRC and CAB (3).

The main localization of D2DR is on normal and tumoral animal and human lactotroph pituitary cells (2, 4, 5), therefore DAs represent the treatment of choice for PRL-secreting PitNET, for whose management, unlike other PitNET, the consensus guidelines have recommended the medical therapy as first-line treatment of choice rather than surgery (6). In prolactinomas, medical therapy relies on the preferential use of CAB compared to BRC because of the higher affinity of CAB to D2DR than BRC, its better tolerability profile, and its higher and long-lasting efficacy in normalizing prolactin (PRL) levels and in inducing tumor shrinkage (6). Indeed, in prolactinomas receiving treatment with CAB biochemical control and relevant tumor shrinkage are reportedly recorded in the vast majority of patients up to 100% and 96% of cases, respectively (7).

The D2DR localization in normal and tumoral animal and human somatotroph (4, 8–10) and normal and tumoral human corticotroph pituitary cells (11, 12) has provided the basis for DAs application also in the therapeutic algorithm of acromegaly and, as off-label use, in Cushing’s Disease (CD). However, in patients harbouring GH-secreting PitNET and ACTH-secreting PitNET, the biochemical and tumoral efficacy of DAs is lower than in prolactinomas. Indeed, DAs, mainly CAB, are indicated as monotherapy in patients with GH-secreting PitNET with mild disease (13), defined as mild signs and symptoms of GH excess and modest elevation in serum IGF-I levels (lower than 2 times the upper limit of normal) (14). In such patients, CAB administration has resulted in the achievement of biochemical control in approximately one-third of patients (15). Alternatively, CAB can be used in combination with somatostatin receptor ligands (SRLs) in patients displaying partial responsiveness to SRLs monotherapy (14), defined as incomplete or inadequate biochemical and tumoral control (16), or in addition to the GH receptor antagonist pegvisomant (17). In such patients, CAB addition to SRLs has resulted in the achievement of biochemical control in 52% of patients (15). When combined with pegvisomant, CAB administration has been demonstrated to induce biochemical control in 68% of patients (17). In CD, DAs are listed among the pituitary-directed drugs for patients with ACTH-secreting PitNET, including those who have experienced a noncurative surgery or a postoperative recurrence and are not candidates for additional pituitary surgery (18). In such patients, the use of CAB has resulted in the achievement of biochemical control in up to 40% of cases (19). Added to other compounds, CAB has been shown to induce biochemical control in 56-79% when used in combination with steroidogenesis inhibitors (20–22) and up to 88% when administered in addition to pasireotide and ketoconazole (23).

Despite the proven biochemical and tumoral efficacy of DAs in prolactinomas, a minority of patients, accounting for 10% of patients with macroprolactinoma and less than 20% of those with macroprolactinoma (24, 25), fail to achieve the biochemical control of PRL excess and/or the reduction in tumor mass during treatment with DAs (6, 26).

In patients with persistent/recurrent clinically nonfunctioning PitNET, for which no medications have been approved, the efficacy of DAs, including CAB and BRC, in reducing the tumor size has been tested based on the D2DR expression established in most of the nonfunctioning PitNET tissues in several preclinical studies (27–29). DA therapy evaluated in some case reports and in small series provided variable results with a reduction in tumor size in 30% of cases and induction of stable disease in 58% of cases (30). However, due to insufficient and variable evidence, DA therapy is not routinely administrated in patients with nonfunctioning PitNET and DAs use is still a matter of debate (31).

The molecular mechanisms of DAs resistance in PitNET are not fully understood. This mini-review focuses on the molecular mechanisms underlying the resistance to DAs in PitNET.

## Dopamine Receptors and Their Regulation of Physiological and Pathophysiological Mechanisms in Pituitary Tumors

DRs are five subtypes of G protein-coupled receptors (GPCRs) divided into two subgroups based on their structural, functional and pharmacological properties: D1-like family, including D1DR and D5DR, and D2-like family, comprising of D2DR, D3DR, and D4DR (2). The presence of introns within the coding regions of D2-like receptors give rise to the potential formation of isoforms due to the occurrence of alternative splicing in their mRNAs (2). Indeed, D2 receptor exists in two different isoforms, denominated D2 short (D2S) and D2 long (D2L) isoforms, differing for the presence or absence of 29 amino acids in the third intracellular loop, and displaying distinct physiological and pharmacological properties (2).

In lactotroph cells, the dopamine pathway, besides the well-known regulation of hormone secretion, controls the complex physiological mechanism of cell homeostasis. The lactotroph cell homeostasis represents a condition of biological and molecular steady-state to prevent the triggering of pathological conditions. Indeed, in lactotroph cells, a correct function of the dopamine signalling pathway is needful to limit PRL synthesis and secretion, and cell growth and proliferation, to minimize the development of lactotroph hyperplasia, or lactotroph differentiation and expansion, with a consequent formation of PRL-secreting tumors leading to a state of pathological hyperprolactinemia (32–37).

DRs are involved in human and animal hormone secretion inducing the cyclic AMP (cAMP) pathway. Precisely, D1-like receptors, being coupled to stimulatory G proteins (Gα_s_), activate cAMP pathway, inducing adenylyl cyclase (AC) activity, cAMP accumulation, protein kinase A (PKA) activation and calcium (Ca^2+^) release from the intracellular compartment. Conversely, D2-like receptors, being coupled to inhibitory G proteins (Gα_i/o_), inhibit cAMP pathway, suppressing AC activity, cAMP accumulation, PKA activation and Ca^2+^ release from the intracellular compartment (2). Interestingly, studies in rodents have demonstrated that D2S and D2L isoforms induce two cAMP-independent pathways in the regulation of lactotroph cell homeostasis: the mitogen-activated protein kinase (MAPK) and the phosphatidylinositol 3-kinase (PI3K)/protein-kinase B (PKB) pathways.

Specifically, studies on mouse lactotroph cell models demonstrate that D2S induces ERK1/2 of the MAPK pathway and PKB of PI3K pathway, whereas D2L inhibits ERK1/2 and PKB. In physiological conditions, the expression of both isoforms is sufficient to maintain PRL synthesis and secretion and cell proliferation, while the simultaneous knockdown of both D2DR isoforms induces the inhibition of ERK1/2 and the induction of PKB activity resulting in uncontrolled cell proliferation and consequent pituitary hyperplasia and hyperprolactinemia (35). Conversely, in pathological states, the expression of only one D2DR isoform is no longer sufficient to control lactotroph homeostasis (35). These data suggest that the control of D2L/D2S ratio and therefore the balance between MAPK and PI3K pathway are a prerequisite to maintaining lactotroph homeostasis and preventing pathological lactotroph development.

More recently, a new mechanism of DRs modulation has been demonstrated to be involved in the regulation of pituitary tumor volume shrinkage. Indeed, in *in vitro* rodent prolactinoma and somatotropinoma cell lines (MMQ and GH3) and *in vivo* rodent pituitary tumors it has been demonstrated that the activation of D5DR mediates cell growth suppression by autophagic pathway stimulation through PKB/mTOR signalling (38–40). Deeply, D5DR activation inhibits mTOR signalling, decreasing p70S6K and 4eBP1 phosphorylations. Consequently, D5DR activation induces autophagic cell death, enhancing the protein expression of the classical hallmark of autophagy activation, the microtubule-associated protein light chain 3-II (LC3-II), but also regulating the levels of several molecules known as hallmarks of apoptosis but also involved in the autophagic process (38, 39, 41). Indeed, D5DR activation increases reactive oxygen species (ROS) levels, rises the cleavage of poly-(ADP-ribose) polymerase (PARP) and the Caspase-3, and decreases the superoxide dismutases (SOD) protein expression (38, 39, 41). Interestingly, BRC and CAB have been found to prompt cell death *via* different pathways; BRC induces prolactinoma cell death mainly through the apoptosis pathway, while CAB induces prolactinoma cell death mainly *via* the autophagic cell death pathway (40).

Interestingly, D2DR has been also demonstrated to be involved in the growth and invasiveness as well as in the growth of stem-like cells, the main source of drug resistance, of nonfunctioning PitNET. Indeed, the activation of D2DR significantly induces antiproliferative effects by activating ERK1/2, p38 MAPK and caspase-3 in primary cultures of nonfunctioning PitNET (42) and induces antiproliferative effects on primary stem-like cell spheres of nonfunctioning PitNET (43). Moreover, the activation of D2DR significantly reduces the migration and invasion of a human cell model and of primary cultures of nonfunctioning PitNET, through the Rho-associated protein kinase (ROCK)-dependent LIMK (LIM kinase, an actin-binding kinase) activation. D2DR activation of ROCK/LIMK pathway determinates the inactivation of the protein cofilin, an actin-binding protein that regulates filament dynamics, with a consequent loss of its ability to bind the actin and, thus, promoting cell migration (44).

## Dopamine Agonists Resistance in Pituitary Tumors

Guideline for hyperprolactinemia has defined the DAs resistance as a failure to achieve normal PRL levels together with a ≥ 50% reduction in tumor size at maximally tolerated doses (6). Only a small subset of patients with prolactinomas does not respond to DAs treatment (primary resistance), as approximately 20-30% is resistant to BRC and around 10-20% to CAB (24, 45), due to modification of DRs expression or to intracellular molecular mechanisms. Very infrequently patients with prolactinomas experience a delayed resistance becoming refractory to DAs prolonged treatment (secondary resistance) (46–51). This condition is generally considered an expression of a negative prognosis as it might indicate a malignant transformation of the prolactinoma (46).

In GH-secreting PitNET, the efficacy of DAs, particularly CAB, is remarkably lower than prolactinomas, regardless of their administration as monotherapy or in addition to other compounds; similarly, in ACTH-secreting PitNET the therapeutic efficacy of DAs, mainly CAB, is limited by a decreased response over time leading to escape to treatment (12, 52, 53).

**Figure 1:**
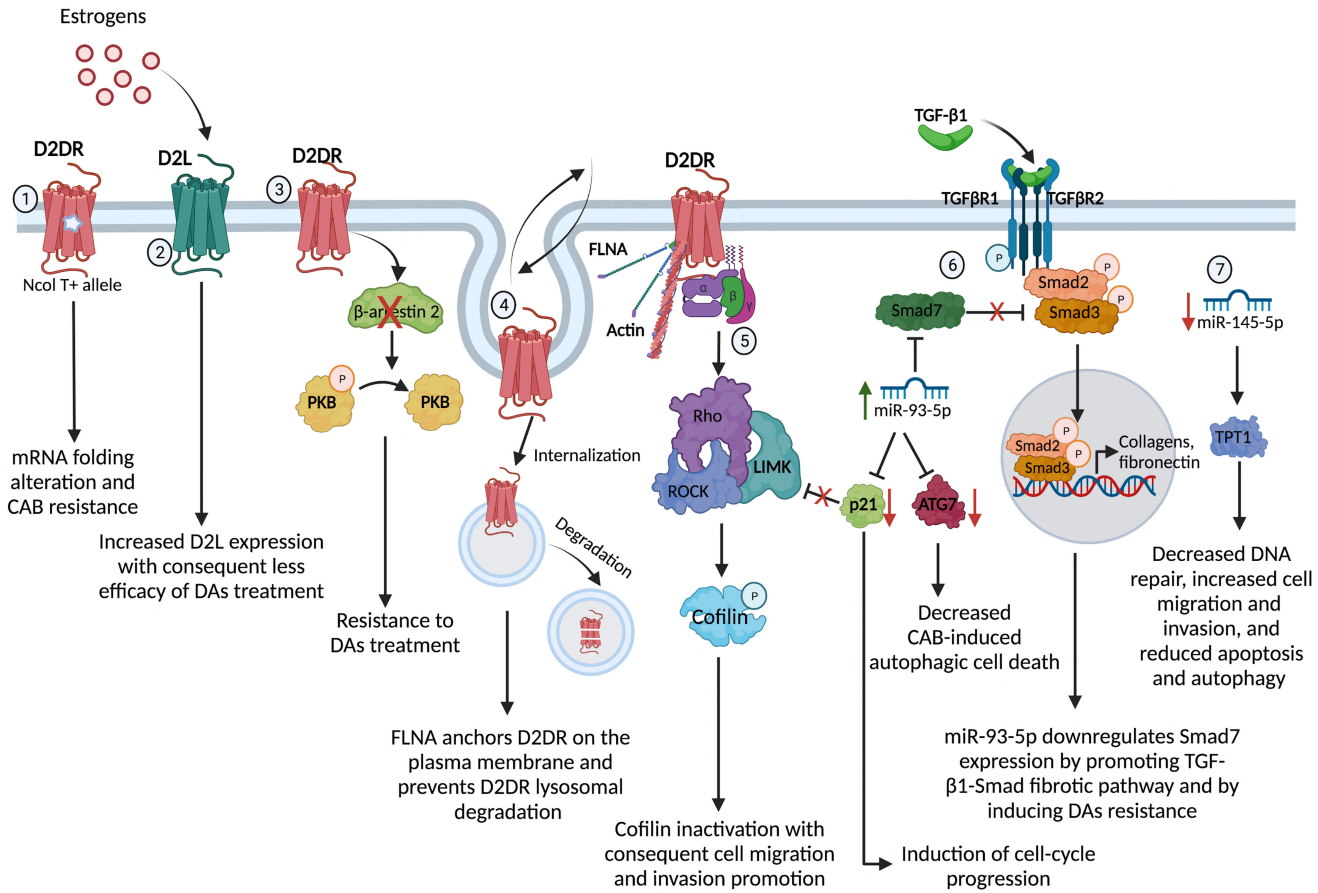
Molecular mechanisms underpinning the DAs resistance in PRL- and GH-secreting PitNET. 1) The gene variant NcoI T+ of the synonymous polymorphism NcoI, consisting in a cytosine to thymine (NcoI C/T) transition at position 957, leads to a decreased D2DR mRNA stability and synthesis through a putative alteration in the receptor mRNA folding, conferring resistance to the antitumoral action of CAB in PRL-secreting PitNET; 2) Estrogens may affect the D2S/D2L ratio tumors, increasing the expression of D2L and therefore affecting the efficacy of DAs treatment in PRL-secreting PitNET; 3) D2DR in PRL-secreting PitNET cell model and primary cultures of nonfunctioning PitNET triggers the β-arrestin 2-mediated PKB dephosphorylation inducing antiproliferative effect and the lack of β-arrestin 2 induces DAs resistance in nonfunctioning PitNET; 4) FLNA is involved in the regulation of D2DR membrane expression and signalling. Indeed, FLNA anchorage and expression of D2DR on the plasma membrane, by controlling D2DR fate towards recycling processes or degradation in PRL- and GH-secreting PitNET; 5) D2DR activates ROCK/LIMK pathway with consequent inactivation of the protein cofilin resulting in a loss of its ability to bind the actin and, thus, promoting cell migration and invasion; 6) miR-93-5p targets Smad7, a negative regulator of TGF-β1/Smad signalling, sustaining the TGF-β1-induced fibrosis in PRL-secreting PitNET. Alongside, miR-93-5p down-regulates p21 inducing cell-cycle progression and losing the control of ROCK/LIMK pathway, and down-regulates ATG7, decreasing the autophagic cell death induced by CAB in PRL- and GH-secreting PitNET; 7) the reduced expression of miR-145-5p stimulates the TPT1 protein resulting in decreased DNA repair, increased cell migration and invasion and reduced apoptosis and autophagy. D2DR, dopamine receptor type 2; PKB, protein-kinase B; FLNA, filamin A; ROCK, Rho-associated protein kinase; LIMK, LIM kinase; ATG7, Autophagy Related 7 protein; TPT1, translationally controlled tumor protein. Created with BioRender.com.

Several molecular mechanisms, reviewed in the following sections and summarized in **Figure 1**, seem to play a role in DAs resistance.

## DRs Expression in Pituitary Tumors: Mechanisms of Resistance

Generally, the effectiveness of BRC or CAB has been related to a decreased expression in DRs, as the expression of such receptors has been shown to correlate with responsiveness to therapy in lactotroph, somatotroph, corticotroph and in clinically nonfunctioning PitNET (12, 54–58). Particularly, a lower messenger expression of D2S and D2S/D2L ratio has been confirmed in the DAs resistant pituitary tumors (12, 27, 28, 54, 56, 59), although in a Japanese study the resistance to DAs in prolactinomas has been surprisingly correlated to a reduced messenger expression of D2L receptor subtype (57). In contrast to these studies, nonfunctioning PitNET response to DAs treatment is not related to D2DR mRNA and protein expression (60, 61), suggesting that other additional factors may mediate the growth inhibitory effects of DAs in nonfunctioning PitNET.

### Modulation of DRs Expression

DRs expression can be modulated by several factors, contributing to affect sensitivity to DAs. Indeed, besides their direct stimulatory role on PRL gene expression and lactotroph mitotic activity (62), estrogens may affect the D2S/D2L ratio tumors, increasing the expression of D2L and therefore affecting the efficacy of DAs treatment, as demonstrated in mouse normal pituitary and pituitary tumor cell lines (63–66). On the contrary, DAs resistant cells, obtained from mice primary lactotroph tumors exposed to nerve growth factor (NGF), have been found to change their phenotype into a differentiated, less malignant lactotroph-like phenotype re-expressing the D2DR protein and recovering the capability to respond to DAs (67).

DRs expression is not the only prerequisite to ensure the efficacy of DAs therapy. Several biological mechanisms, including DRs genetic alterations, receptors desensitization, internalization and intracellular trafficking, and microRNA (miRNA) expression levels can modulate the success of DAs treatment of the pituitary tumors.

### D2DR Genetic Alterations Associated With Resistant Pituitary Tumor

CAB resistance in prolactinomas has been significantly associated with the presence of a D2DR polymorphism. The polymorphism recognized by the restriction enzyme NcoI consists of a synonymous cytosine to thymine (NcoI C/T) transition at position 957. The gene variant NcoI T+, rather than being silent, leads to a decreased D2DR mRNA stability and synthesis through a putative alteration in the receptor mRNA folding, conferring resistance to the antitumoral action of CAB (68). Presently, neither mutations in D2DR gene nor methylated sites in D2DR promoter region have been found to be associated with resistance to DAs treatment in pituitary tumors.

### D2DR Heterodimerization With Somatostatin Receptors

Heterodimerization between D2DR and somatostatin receptor (SSTR) type 2 (SSTR2) and/or type 5 (SSTR5) (69) induces a modification of the ligand-binding and a synergistic effect on the activation of transduction pathways that can change the response to DAs and SRLs. Based on this rationale, chimeric agonists have been produced for therapeutic use, to be used to overcome the DAs and SRLs resistance.

Interestingly, the chimeric compound BIM-23A760, binding with high affinity SSTR2>SSTR5 and D2DR, has been found to regulate hormonal release, exerting both anti- and pro-secretive effects in human GH-secreting, ACTH-secreting, and nonfunctioning PitNET through the regulation of free cytosolic calcium levels (70, 71) and antiproliferative and/or pro-apoptotic effects in *in vitro* human GH-secreting, PRL-secreting and nonfunctioning PitNET (42, 70, 72, 73), in these latter by activating ERK1/2 and p38 pathways and caspase-3 (42). Contrary to what is observed in GH-secreting PitNET where the chimeric compound BIM-23A760 produced a greater GH suppression in partial responder tumors to SRL octreotide (71), in nonfunctioning PitNET the effect exerts from chimeric compound BIM-23A760 is not significantly different from that triggered by the specific DA (42, 73). Indeed, in nonfunctioning PitNET the efficacy of the chimeric compound BIM-23A760 is mainly dependent on D2DR activation (73), although no correlation has been found between D2DR expression and the sensitivity to BIM-23A760 in this pituitary tumor model (73).

Interestingly, the chimeric compound BIM-23A760 has been found to significantly reduce the cell viability of stem-like cell subsets of different pituitary tumors (74), thus representing potential novel therapeutic agents for therapy-resistant tumors. Despite this, in primary cultures of nonfunctioning PitNET the D2DR agonist BIM-53097 has been shown to reduce the cell viability of stem-like cells as well (43), but given the lack of evidence comparing the selective D2DR agonist with the chimeric compounds in the regulation of stem-like cells cell viability in nonfunctioning PitNET, it cannot be stated that the mutual activation of several receptors through chimeric compounds is sufficient to overcome the pharmacological resistance observed in some resistant nonfunctioning PitNET. Up to now, the lack of biological and clinical determinants still leaves unresolved the question of the molecular mechanisms underlying the resistance to DAs in nonfunctioning PitNET.

### The Role of Cytoskeleton Protein Filamin-A

Like other GPCRs, DRs ensure the appropriate magnitude and duration of the extracellular stimuli translation into intracellular signals *via* three main modes of regulation: desensitization, a process in which a receptor becomes refractory to continued stimuli; internalization, a process in which receptors are physically removed from the cell surface by endocytosis; and down-regulation, a process in which total cellular receptor levels are decreased. In the last years, the cytoskeleton protein Filamin-A (FLNA), an actin-binding protein that regulates reorganization of the actin cytoskeleton by interacting with integrins, several GPCRs, ion channels and second messengers anchoring them to the actin cytoskeleton, is associated with the regulation of D2DR expression and signalling (75–77) in several cell system including pituitary tumor cells (78, 79). In melanoma cell models, the expression of FLNA allows the plasma membrane localization of D2DR, whereas, in absence of FLNA, D2DR is predominantly localized in the cytoplasm compartment (76). Consistently, FLNA protein expression has been found reduced in DAs resistant human prolactinomas and FLNA silencing by small interfering RNA in DAs sensitive human prolactinomas has been shown to result in a reduced protein expression of D2DR, abrogation of PRL secretion inhibition and antiproliferative signals (78), demonstrating the crucial role of FLNA in D2DR expression and its role as scaffold for signalling molecules involved in D2DR signal transduction in lactotroph tumors. Moreover, experiments conducted on the rodent prolactinoma cell line MMQ have demonstrated that FLNA not only allows the anchorage and expression of D2DR on the plasma membrane, but it also prevents D2DR lysosomal degradation (78), demonstrating that FLNA may control D2DR fate towards recycling processes or degradation. FLNA expression has been also correlated to D2DR mRNA expression in GH-secreting PitNET (79).

### The Role of β-Arrestins

The activation of DRs, particularly of D2DR, by DAs is quickly followed by their rapid phosphorylation induced by GPCR kinases (GRK) and protein kinase C (PKC) (80–83). This results in the recruitment of β-arrestin 1 and 2, multifunctional scaffolding proteins, involved in desensitization and internalization as well as in the induction of signal transduction protein complexes, in which β-arrestin acts as a scaffold for different kinases and phosphatases, of several GPCRs, including D2DR (84–86). However, D2DR desensitization and resensitization have been reported to be also mediated by β-arrestin in a phosphorylation-independent manner (87, 88).

β-arrestins have been demonstrated to be expressed in human pituitary tumors, including lactotroph, somatotroph, corticotroph and clinically nonfunctioning PitNET (88–90), with a higher expression of β-arrestin 2 compared β-arrestin 1 (88, 89). Interestingly, β-arrestin 2 is expressed more frequently in nonfunctioning PitNET responsive to the D2DR-selective agonist BIM53097 (90).

Moreover, β-arrestins are expressed in rodent MMQ and GH3 cell lines, β-arrestin 2 being the only one expressed in the MMQ cell line (90) and the most highly expressed in GH3 cell line as compared to β-arrestin 1 (89). Remarkably, β-arrestin 2 is not only involved in the regulation of D2DR expression but also the regulation of its signal transduction pathway. Indeed, it has been shown that the BIM53097 agonist binding to D2DR in MMQ in primary cultures of nonfunctioning PitNET triggers the β-arrestin 2-mediated PKB dephosphorylation inducing antiproliferative effect (90). These data have been corroborated by experiments conducted on primary cultures of nonfunctioning PitNET lacking β-arrestin 2 expression and not responsive to DAs. Plasmid transfection of β-arrestin 2 in such cultures restored the ability of D2DR-selective agonist BIM53097 to inhibit cell proliferation (90), demonstrating that β-arrestin 2 has a main role in the responsiveness to DAs in pituitary tumors.

### The Role of microRNAs

microRNAs (miRNAs) are endogenous, single-strand, highly conserved, small non-coding RNAs with a length of approximately 22–25 nucleotides (91). miRNAs play a significant role in gene expression regulation, inducing mRNAs degradation or repressing protein synthesis through the binding to seed sequences located in the 3’-untranslated regions (3′-UTR) or the 5’-untranslated regions (5′-UTR) or the coding region of their target mRNAs, thus silencing the target genes. In particular, miRNAs binding to the sites located in the coding regions are more potent in inhibiting translation, while, binding to the sites located in the 3′-UTR are more efficient in triggering mRNA degradation (91–94).

Recent evidence has revealed that several miRNAs are involved, among other functions, in regulating drug resistance in multiple tumors, including pituitary tumors (95–99).

miR-93, miR-17, miR-22, miR-126, miR-142-3p, miR-144, miR-486-5p, miR-451 and miR-92a were up-regulated while miR-30a, miR-382, miR-136 have been found down-regulated in BRC-resistant prolactinomas compared to BRC-sensitive prolactinomas (97). Interestingly, the knockdown of endogenous miR-93-5p by the miRNA inhibitor antagomir transfection in MMQ cells has been shown to significantly increase the sensitivity to BRC and CAB treatment inducing cell proliferation inhibition, whereas overexpression of miR-93-5p with the miRNA mimic agomir transfection blunted the anti-secretive effect of BRC on PRL release (95) and suppressed the cytotoxic effect of CAB in MMQ and GH3 cells (97, 99). The DAs resistance induced by the overexpression of miR-93-5p in MMQ and GH3 cell lines involves the down-regulation of the protein p21, a key member of cyclin kinase inhibitors known to be implicated in the inhibition of cell-cycle progression, as demonstrated in MMQ cell line (97), and the down-regulation of the protein Autophagy Related 7 (ATG7), an essential regulator of autophagy, decreasing the autophagic cell death induced by CAB in MMQ and GH3 cell lines and rat pituitary tumors (99).

About 43% of the DA-resistant prolactinomas have been reported to be highly fibrotic and to have a higher collagen content compared to the DA-responsive ones (100). As observed in other tumors, the initiation and development of tissue fibrosis in prolactinomas is mediated by TGF-β1/Smad3 pathway and the expression of TGF-β1/Smad3 signalling pathway components be elevated in DA-resistant and fibrotic prolactinomas (100). Remarkably, miR-93-5p is highly expressed in DA-resistant prolactinomas with a high degree of fibrosis (95). The *in vitro* study performed in primary cultures of human prolactinomas has revealed that TGF-β1 increases the expression of miR-93-5p, which in turn targets Smad7, a negative regulator of TGF-β1/Smad signalling. The blunting of Smad7 expression by miR-93-5p promotes the DAs resistance sustaining the TGF-β1-induced fibrosis in prolactinoma cells (95).

More recently, decreased miR-145-5p levels have been shown in BRC-resistant human prolactinomas and in BRC-resistant MMQ cell line with a concomitant higher expression of the translationally controlled tumor protein (TPT1), a protein that plays a crucial role in many biological processes including DNA damage repair, epithelial to mesenchymal transition (EMT), migration, invasion, apoptosis and autophagy by interacting with several other proteins (96).

### Other Molecular Mechanisms

Adequate tumor vascularization, defined by the acquisition of angiogenic phenotype, is a prerequisite for the further outgrowth of the tumor, as observed in several human tumors including pituitary tumors (101). Macroprolactinomas have been found to display a higher degree of vasculature than microprolactinomas, and similarly, invasive pituitary prolactinomas have been reported to be significantly vascularized (101). The vascular endothelial growth factor (VEGF), the hallmark and central mediator of angiogenesis, has been reported to be highly expressed in DAs resistant prolactinomas (102, 103). Indeed, the dopaminergic system, mediated through the D2DR, negatively regulates the angiogenesis, inhibiting the vascular permeabilizing and angiogenic activities of VEGF (104), as also demonstrated in D2DR knockout mice models, in which mRNA and protein expression of pituitary VEGF-A are increased compared with wild-type mice, demonstrating that pituitary VEGF expression is under dopaminergic control (105).

## Conclusions

In conclusion, the pharmacological efficacy of DAs used for the medical treatment of PitNETs is based on several multifaceted mechanisms, mainly investigated in prolactinomas. These mechanisms include the reduction of D2DR expression, regulated by internalization and down-regulation processes orchestrated by cytoskeleton proteins and β-arrestins; changes in the proportion of D2DR subtypes; the regulation of cell signalling pathway of D2DR; and the increase of angiogenic and fibrotic markers. Growing evidence has highlighted the role of miRNAs in DAs resistance, particularly through the regulation of fibrotic pathway, although further studies are required to better elucidate the burden and the role of molecular mechanisms underlying pharmacological resistance to DAs. 

## Author Contributions

CP conceived and wrote the manuscript. RPa, MN, and RPir contributed to the literature search and manuscript preparation. AC and RPiv provided a significant expert contribution to the scientific revision process. RSA supervised the manuscript drafting and critically reviewed the content. All authors contributed to the article and approved the submitted version.

## Funding

This work was supported by the Ministry of Education, University and Research Grants PRIN 2017S55RXB (to RSA) and by POR FESR CAMPANIA 2014–2020 “Diagnostic and therapeutic innovations for rare neuroendocrine, endocrine tumors and glioblastoma by an integrated technological platform of clinical, molecular, genomic, ICT, pharmacological and pharmaceutical expertise” RARE.PLAT.NET (to AC).

## Conflict of Interest

RPiv has been the principal investigator of Clinical and/or Translational Research Studies for Novartis, HRA Pharma, Ipsen, Shire, Corcept Therapeutics, Cortendo AB-Strongbridge Biopharma, Janssen Cilag, Camurus and Pfizer; Co-investigator of Research Studies for Pfizer; received research grants from Novartis, Pfizer, Ipsen, HRA Pharma, Shire, IBSA, Strongbridge Biopharma; has been an occasional consultant for Novartis, Ipsen, Pfizer, Shire, HRA Pharma, Cortendo AB-Strongbridge Biopharma, Ferring, Recordati Rare Disease, Corcept Therapeutics, Crinetics Pharmaceuticals, ARH Healthcare, Biohealth Italia, Damor Farmaceutici; and has received fees and honoraria for presentations from Novartis, Shire, Pfizer and Recordati beyond the confines of this work. CP received research grants from Corcept Therapeutics. AC has been the principal investigator of Research Studies for Novartis, Ipsen, Pfizer, Lilly, Merck and Novo Nordisk; consultant for Novartis, Ipsen, Pfizer, and received honoraria from Novartis, Ipsen and Pfizer beyond the confines of this work.

The remaining authors declare that the research was conducted in the absence of any commercial or financial relationships that could be construed as a potential conflict of interest.

## Publisher’s Note

All claims expressed in this article are solely those of the authors and do not necessarily represent those of their affiliated organizations, or those of the publisher, the editors and the reviewers. Any product that may be evaluated in this article, or claim that may be made by its manufacturer, is not guaranteed or endorsed by the publisher.
